# Phylogenetic Beta Diversity Metrics, Trait Evolution and Inferring the Functional Beta Diversity of Communities

**DOI:** 10.1371/journal.pone.0021264

**Published:** 2011-06-24

**Authors:** Nathan G. Swenson

**Affiliations:** Department of Plant Biology, Michigan State University, East Lansing, Michigan, United States of America; University of Zurich, Switzerland

## Abstract

The beta diversity of communities along gradients has fascinated ecologists for decades. Traditionally such studies have focused on the species composition of communities, but researchers are becoming increasingly interested in analyzing the phylogenetic composition in the hope of achieving mechanistic insights into community structure. To date many metrics of phylogenetic beta diversity have been published, but few empirical studies have been published. Further inferences made from such phylogenetic studies critically rely on the pattern of trait evolution. The present work provides a study of the phylogenetic dissimilarity of 96 tree communities in India. The work compares and contrasts eight metrics of phylogenetic dissimilarity, considers the role of phylogenetic signal in trait data and shows that environmental distance rather than spatial distance is the best correlate of phylogenetic dissimilarity in the study system.

## Introduction

Determining the relative importance of the factors influencing the diversity and turnover of ecological communities along gradients has been a persistent theme in ecology [1]. Increasingly ecologists have turned to phylogenetically- and functionally-based investigations of communities in order to provide more detailed information regarding the species in their study systems [2]–[10]. In the tropics, phylogenetic investigations have become particularly popular when quantifying the multi-dimensional functional strategy of hundreds of co-existing species may be unfeasible [2], [5]. These studies have focused on quantifying whether the phylogenetic diversity in an assemblage is higher or lower than that expected given the observed species richness and a species pool. Using the assumption that phylogenetic relatedness is positively correlated with ecological or functional similarity, such studies have made inferences regarding the role of abiotic and biotic interactions in structuring communities.

In recent years, community ecologists have expanded the above phylogenetic approach to include analyses of phylogenetic beta diversity or turnover between communities [11]–[15]. This approach is potentially powerful in that it can detect phylogenetically basal or terminal turnover between communities that traditional species-based metrics do not. For example, the turnover of con-geners along an environmental gradient would be considered low phylogenetic turnover, but high species turnover. These opposing patterns may have substantial consequences for how we understand the structure of communities [14]. Despite the power of this phylogenetic beta diversity approach, few empirical examples exist and to my knowledge there are no existing examples from diverse tropical ecosystems. In particular, we do not know whether spatial distance or environmental distance is more correlated with the phylogenetic turnover between communities in diverse systems like tropical tree communities. For example, ancient divergences in habitat preferences between clades and little divergence within clades should generate high levels of phylogenetic beta diversity along environmental gradients whereas recent large habitat shifts should provide the opposite pattern. Thus instead of simply knowing that species composition turns over along environmental gradients, phylogenetic metrics can begin to provide insights into how the evolution of habitat preferences or species function has influenced the observed distributional patterns.

Although analyses of phylogenetic diversity within and between communities are potentially very powerful particularly in diverse ecosystems, they both critically rely on the assumption that phylogenetic relatedness is a sound proxy for functional or ecological similarity. This assumption is routinely questioned and examples where the assumption is violated are not difficult to find particularly when examining patterns of trait evolution [16]–[18]. Thus phylogenetic community ecologists are tasked with quantifying the phylogenetic signal in trait data rather than assume that it is there in order to make robust inferences [3], [19]. In particular, if there is phylogenetic signal in trait data, then the patterns of phylogenetic diversity in a community or between communities should mirror the functional diversity [3]. Simulation-based studies that have examined alpha diversity have generally supported this expectation [20], but similar studies of phylogenetic beta diversity have not been conducted. In particular, it is not clear whether the functional beta diversity of communities can be predicted from the phylogenetic beta diversity when there is, or is not, phylogenetic signal in functional trait data. For example, how much phylogenetic signal is needed for a phylogenetic beta diversity metric to accurately recover the functional beta diversity of two communities?

Another challenge for phylogenetic analyses of beta diversity is the rapid accumulation of metrics that may or may not be redundant. Thus it will become increasingly difficult to compare and contrast the results across different studies and to determine which metrics provide novel information over others. The present study utilizes a large tree inventory plot dataset from India to address the above outstanding challenges for investigations into the phylogenetic dissimilarity of communities. Specifically, here I ask: (*i*) is spatial or environmental distance more correlated with the phylogenetic beta diversity of tropical tree communities?; (*ii*) how much phylogenetic signal in trait data is needed for phylogenetic beta diversity metrics to reflect the functional beta diversity and how does this vary from metric to metric?; and (*iii*) are any of the eight phylogenetic beta diversity metrics used in this study redundant and which provide novel insights? The second and third questions are largely of a methodological nature, but answering these questions is critical for one to appropriately address the first question posed. That is, without addressing the statistical underpinnings and relationships of the large number of phylogenetic beta diversity metrics that are accumulating it is difficult, if not irresponsible, to address the biological questions of interest with these metrics. Thus, the work will primarily focus on the key methodological questions while trying to provide some biological insights along the way.

**Figure 1:**
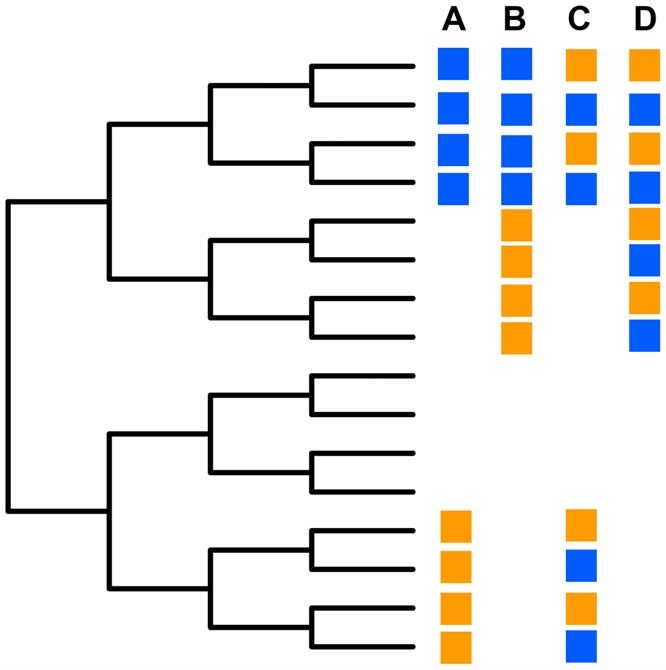
An example of four pairs of hypothetical communities and types of phylogenetic beta diversity. The species in a single community have the same color boxes. Species that are in neither community are left blank. All branch lengths are set to one and all species are scored as present or absent in this simplified example. It is important to note that in each of the four scenarios there is a complete turnover of species between the two communities, but the degree of phylogenetic beta diversity varies. Scenario A indicates species in the blue community are closely related to one another, but distantly related to the species in the orange community. This is an example of ‘basal’ phylogenetic turnover. Scenario B also indicates species in the blue community are closely related to one another, but distantly related to the species in the orange community. The main difference in that Scenario B has a much lower level of ‘basal’ phylogenetic beta diversity than that in Scenario A. Scenario C indicates locally phylogenetically overdispersed communities that have little phylogenetic beta diversity. Scenario D also indicates local phylogenetic overdispersion and low phylogenetic beta diversity. In both scenarios phylogenetic beta diversity measured using a nearest neighbor metric will be lower than when measured using a pairwise metric that considers the basal portion of the phylogeny and this effect will be maximized in Scenario C.

Several metrics of phylogenetic beta diversity have been produced in recent years. In Figure 1 I present a simplified picture of different types of phylogenetic beta diversity or turnover where phylogenetic beta diversity is relatively ‘basal’ or ‘terminal’. In this hypothetical set of scenarios the species turnover between the communities being compared is complete or in other words species beta diversity is the maximum possible. In contrast the phylogenetic beta diversity is more variable.

The present work seeks to analyze eight of the most commonly implemented metrics. There are undoubtedly alternative metrics that have been developed or that will be developed, but for the time being the manuscript will be constrained to the follow set of eight.

The first metric I used is phylogenetic analog of Sorensen's Index termed *PhyloSor* [15]:
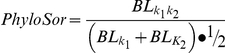
where *BL_k1k2_* is the total length of the branches shared between community *k_1_* and *k_2_*, *BL_k1_* and *BL_k2_* are the total branch lengths found in communities *k_1_* and *k_2_* respectively. This metric may be considered a ‘basal’ metric upon initial inspection, but in reality most of the variability in values necessarily comes from the terminal aspects of the phylogeny unless communities turnover over almost entirely between very basal clades, but this is likely never occurring.

The second metric used is a presence-absence weighted dissimilarity metric representing the unique fraction (*UniFrac*) of the phylogeny represented between two communities [12]:
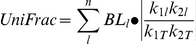
where *n* is the number of branches in the phylogeny, *BL_i_* is the length of branch *l*, *k_1l_* and *k_2l_* are the numbers of species descendent from branch *l* in communities *k_1_* and *k_2_*. Lastly *k_1T_* and *k_2T_* are the total numbers of species in communities *k_1_* and *k_2_* respectively. Similar, to the *PhyloSor* metric this metric primarily will detect ‘terminal’ phylogenetic beta diversity.

The third metric used is presence-absence weighted and calculates the mean nearest phylogenetic neighbor between two communities [21]:
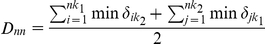
where min 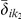 is the nearest phylogenetic neighbor to species *i* in community *k_1_* in community *k_2_* and min 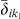 is the nearest phylogenetic neighbor to species *j* in community *k_2_* in community *k_1_.* This metric like those above is a ‘terminal’ metric of phylogenetic beta diversity.

The fourth metric is similar to the above nearest neighbor metric except that it is abundance weighted [21,21]:
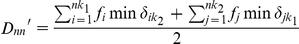
where *f_i_* and *f_j_* are the relative abundance of species *i* and species *j*. This metric like those above is a ‘terminal’ metric of phylogenetic beta diversity.

The fifth metric is a presence-absence weighted pairwise phylogenetic dissimilarity metric [21]:
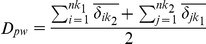
where 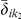 is the mean pairwise phylogenetic distance between species *i* in community *k_1_* to all species in community *k_2_* and 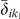 is the mean pairwise phylogenetic distance between species *j* in community *k_2_* to all species in community *k_1_*. This metric unlike those above is a ‘basal’ metric of phylogenetic beta diversity.

The sixth metric is an abundance weighted version of the above pairwise phylogenetic dissimilarity [21], [22]:
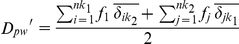
where *f_i_* and *f_j_* are the relative abundance of species *i* and species *j*. This metric can be considered a ‘basal’ metric of phylogenetic beta diversity.

The seventh metric is derived from Rao's quadratic entropy [13], [23]:
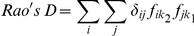
where the variables are the same as those used the above nearest neighbor and pairwise metrics. This metric can be considered a ‘basal metric of phylogenetic beta diversity.

The final metric standardizes Rao's D based upon differences in alpha diversity between the two communities:
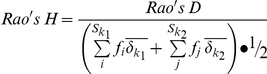
where 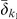 is the mean pairwise phylogenetic distance between species in community *k_1_* and 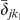 is the mean pairwise phylogenetic distance between species in community *k_2_*. This metric can be considered a ‘basal’ metric of phylogenetic beta diversity.

In the above I describe the eight metrics as relatively ‘terminal’ or ‘basal’ metrics. To demonstrate this property I have calculated each of the presence-absence weighted metrics using the four simplified scenarios showed in Figure 1. I performed the calculations on the original tree in Figure 1 and on four transformed versions of that tree using a lambda transformation [24]. The last of these transformations generated a star phylogeny, which allowed for the comparison of the metrics when all species are equally related. The results in Table 1 provide initial insights into the similarity of some of the metrics and their ability to detect terminal versus basal phylogenetic turnover. In general the nearest neighbor metrics of Dnn, PhyloSor and UniFrac were able to detect terminal turnover in Scenarios C and D and contrast them with the basal turnover in Scenarios A and B. The pairwise metrics of Dpw, Rao's D and Rao's H were able to do the same except the magnitude of the beta diversity measured was the inverse of that for the terminal metrics. This suggests that these two classes of metrics are complementary, rather than redundant, and may be utilized to differentiate patterns such as Scenario C versus D.

**Table 1:** Calculated values for the four scenarios provided in Figure 1 using the six presence-absence weighted metrics used in the article.

Metric	Scenario Letter	λ = 1	λ = 0.75	λ = 0.50	λ = 0.25	λ = 0.001
Dpw	A	8	8	8	8	8
	B	6	6.5	7	7.5	7.998
	C	5.5	6.125	6.75	7.375	7.9975
	D	4.5	5.375	6.25	7.125	7.9965
Dnn	A	8	8	8	8	8
	B	6	6.5	7	7.5	7.998
	C	2	3.5	5	6.5	7.994
	D	2	3.5	5	6.5	7.994
PhyloSor	A	0	0	0	0	2.22E-16
	B	0.125	0.075	0.0417	0.0179	6.25E-05
	C	0.6667	0.4615	0.2857	0.1333	0.0005
	D	0.6364	0.4286	0.2593	0.1186	0.0004
UniFrac	A	1	1	1	1	1
	B	0.9333	0.9610	0.9787	0.9910	0.9999
	C	0.5	0.7	0.8333	0.9286	0.9997
	D	0.5333	0.7273	0.8511	0.9369	0.9998
Rao's D	A	4	4	4	4	4
	B	3	3.25	3.5	3.75	3.999
	C	2.75	3.0625	3.375	3.6875	3.9988
	D	2.25	2.6875	3.125	3.5625	3.9983
Rao's H	A	2.75	2.3125	1.875	1.4375	1.0018
	B	1.75	1.5625	1.375	1.1875	1.00078
	C	0.25	0.4375	0.625	0.8125	0.9993
	D	0.25	0.4375	0.625	0.8125	0.9993

Further the original phylogeny (λ = 1) was lambda transformed [24] four times to produce phylogenies that were increasingly ‘tippy’ ending with a ‘star’ phylogeny where all species are equally related. This simplified example highlights the similarity or redundancy of some of the phylogenetic beta diversity metrics utilized. It also shows that the metrics converge as the phylogeny becomes more ‘star-like’ at which point very little phylogenetic information is available.

The results in Table 1 also show the behavior of the metrics when the phylogeny becomes more ‘star-like’. In particular, each metric converged on a single value across all four scenarios when a star phylogeny was utilized. In other words the phylogenetic metric could not tell the scenarios apart because all species are equally related and every scenario demonstrates maximum phylogenetic turnover. This is intuitive as phylogenetic relatedness is equal between all species and no additional information regarding similarity can be gleaned from this phylogeny. This elucidates the fact that the phylogenetic beta diversity metrics when utilized on a star phylogeny are essentially the same as most species beta diversity metrics. For example, a presence-absence metric like PhyloSor will converge on a traditional Sorensen's Index and an abundance-weighted metric like Dpw will converge on a traditional Bray-Curtis Distance when a star phylogeny is used. Thus nearly all information is lost when a star phylogeny is utilized and this is particularly so for metrics scales between zero and one such as UniFrac and PhyloSor. Metrics that are not scaled between zero and one do provide additional information above and beyond what can be gleaned from a traditional species beta diversity metric in that they still relay branch length information in the form of the distances from the root to the tips of the tree. Whether this information is actually useful for inferences regarding community structure and assembly is another question. Lastly, it should be noted that when the phylogeny was very ‘tippy’ (I.E. lambda  =  0.25) signifying an early burst of speciation followed by stasis, the metrics were still able to differentiate between the scenarios. Thus in the unlikely scenario of a star phylogeny the present metrics of phylogenetic beta diversity may convey little additional useful information to the ecologist, but even in scenarios where there was a rapid radiation followed by little net diversification the metrics still can differentiate between the patterns of importance to the ecologist.

## Results

The first goal of this study was to quantify the relationship between the phylogenetic beta diversity of tropical tree communities and their spatial distance or climatic difference. The results of the Mantel tests show that species and phylogenetic beta diversity was generally more correlated with differences in annual precipitation rather than changes in altitude or spatial distance (Table 2). When comparing the phylogenetic metrics, pairwise metrics *Dpw*, *Dpw*', Rao's D, and Rao's H generally had weaker correlations with annual precipitation differences than did *PhyloSor*, *UniFrac*, *Dnn*, and *Dnn*' (Table 2). These results were consistent whether randomly resolved or the original less well resolved phylogeny was utilized (Table 2).

**Table 2:** The results of Mantel tests used to determine the correlation between community beta diversity metrics and geographic, altitudinal or precipitation differences.

Metric	Species or Phylogenetic	Geographic Distance	Altitudinal Difference	Precipitation Difference
Jaccard	Species	0.070	0.026	**0.194**
Bray-Curtis	Species	0.073	0.026	**0.214**
PhyloSor	Phylogenetic	−0.078 (−0.080–−0.075)	−0.010 (−0.011–−0.008)	**−0.311** (−0.313–−0.308)
UniFrac	Phylogenetic	0.080 (0.077–0.082)	0.013 (0.011–0.015)	**0.300** (0.298–0.302)
Dnn	Phylogenetic	0.068 (0.066–0.071)	0.006 (0.004–0.008)	**0.230** (0.227–0.232)
Dnn'	Phylogenetic	0.083 (0.081–0.085)	0.018 (0.016–0.021)	**0.281** (0.278–0.284)
Dpw	Phylogenetic	0.031 (0.030–0.032)	0.103 (0.101–0.105)	**0.110** (0.107–0.112)
Dpw'	Phylogenetic	0.013 (0.011–0.014)	0.085 (0.083–0.088)	**0.128** (0.126–0.129)
Rao's D	Phylogenetic	0.013 (0.011–0.015)	0.085 (0.082–0.087)	**0.128** (0.126–0.130)
Rao's H	Phylogenetic	0.029 (0.028–0.031)	0.011 (0.008–0.013)	**0.053** (0.052–0.055)

The values in the cells are *r* values and boldface indicates significance with phylogenetic values being calculated from the Phylomatic phylogeny. Values in the parentheses indicate 95% confidence intervals generated from the 100 randomly resolved phylogenies.

**Figure 2:**
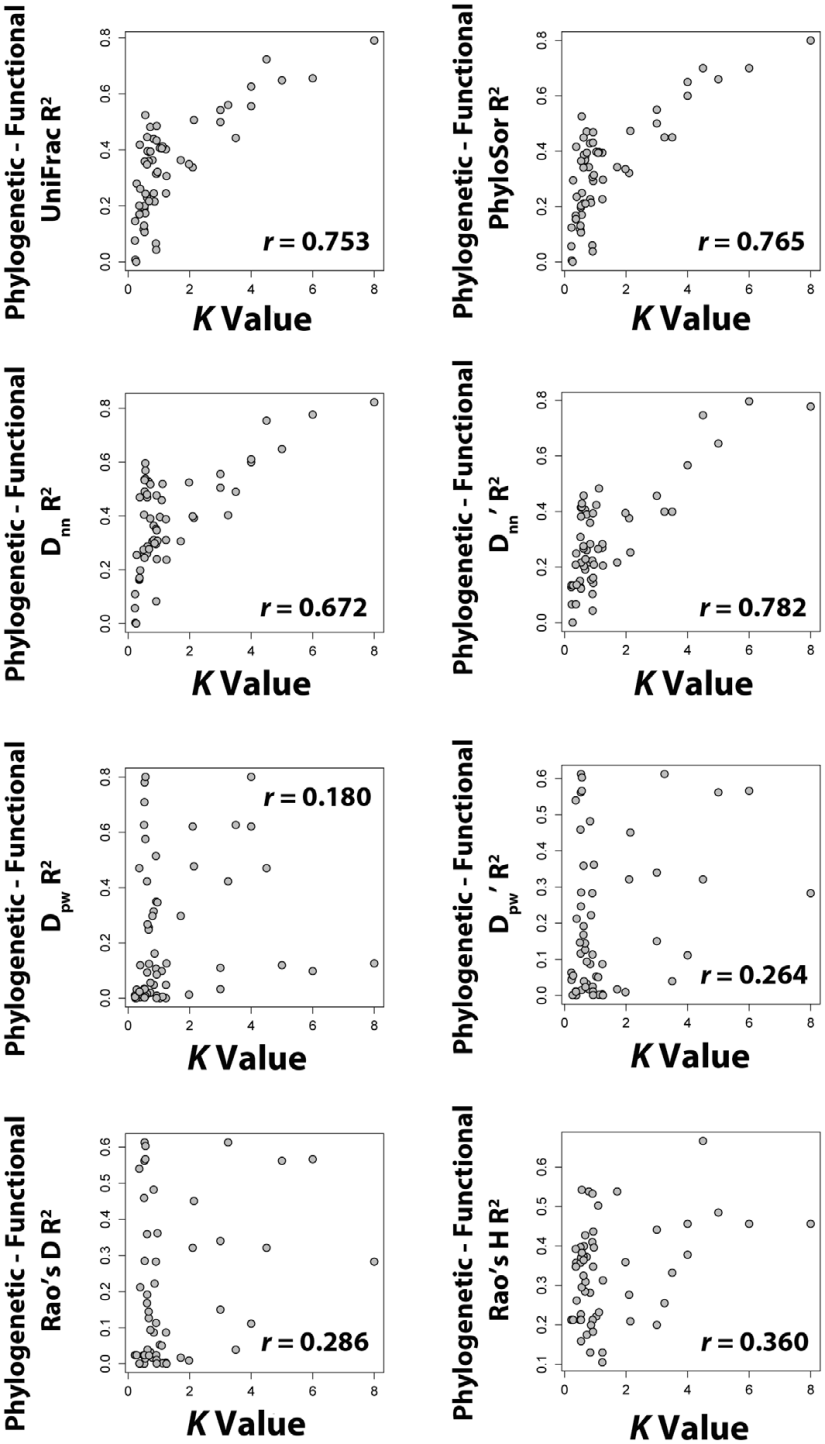
The relationship between phylogenetic signal in trait data (x-axis) and the relationship between the phylogenetic and functional beta diversity of communities (y-axis). Larger *K* values indicate more phylogenetic signal in trait data and higher y-axis values indicate that the phylogenetic beta diversity of the tree plots is more correlated with the functional beta diversity.

The second goal of this study was to examine the relationship between different patterns of trait evolution and the ability to predict functional beta diversity from patterns of phylogenetic beta diversity. The prediction was that a high degree of phylogenetic signal in trait data should strengthen the correlation between phylogenetic and functional beta diversity values. This prediction was supported when using the *PhyloSor*, *UniFrac* and nearest neighbor metrics where the stronger the phylogenetic signal in trait data (i.e. a higher *K* value) the stronger the correlation between the phylogenetic and functional beta diversity patterns (Figure 2). Conversely the pairwise and Rao metrics were less likely to accurately predict the pattern of functional beta diversity even when there was moderate to high phylogenetic signal in the trait data.

A final goal of the present study was to examine the statistical relationships between the eight phylogenetic beta diversity metrics. This was done by calculating Pearson's correlations between the outputs from all metrics and using a principal components analysis. The correlation analyses show strong correlations between many pairs of metrics with some metrics being essentially identical (Table 3). For example, the *PhyloSor* and *UniFrac* metrics are nearly indistinguishable (*R^2^*  =  0.991) and unsurprisingly *Rao*'*s D* and *D_pw_*' were identical (*R^2^*  =  1.00). The two nearest neighbor metrics were generally highly correlated with the *PhyloSor* and *UniFrac* metrics. These metrics were less well correlated with the pairwise and *Rao*'*s D* metrics. The *Rao*'*s H* metric was correlated with both of these groups of metrics, but more strongly with the nearest neighbor metrics. The principal components analysis supported the regression analyses by showing that the nearest neighbor, *PhyloSor* and *UniFrac* metrics loaded heaviest on the first two principal component axes while the pairwise and *Rao*'*s D* metrics only loaded heavily on the third principal component axis (Table 4).

**Table 3:** A correlation analysis of different metrics of species and phylogenetic community dissimilarity.

	Jaccard	Bray-Curtis	PhyloSor	UniFrac	Dnn	Dnn'	Dpw	Dpw'	Rao's D	Rao's H
**Jaccard**	-	-	−0.59–−0.55	0.592–0.598	0.429–0.434	0.592–0.597	0.170–0.178	0.343–0.348	0.344–0.349	0.280–−284
**Bray-Curtis**	0.988	-	−0.626–−0.622	0.642–0.646	0.478 – 0.482	0.650 – 0.656	0.183 – 0.188	0.342 – 0.347	0.343 – 0.349	0.108 – 0.113
**PhyloSor**	−0.57	−0.624	-	−0.993–−0.989	−0.905 –−0.902	−0.805 –−0.801	−0.116 –−0.112	−0.155 –−0.147	−0.155 –−0.150	−0.366 –−0.362
**UniFrac**	0.596	0.644	−0.991	-	0.869 –0.874	0.775 –0.779	0.106 –0.109	0.149 –0.153	0.149 –0.153	0.342 –0.347
**Dnn**	0.431	0.48	−0.904	0.872	-	0.765 –0.770	0.063 –0.061	0.074 –0.078	0.073 –0.077	0.465 –0.472
**Dnn'**	0.594	0.653	−0.803	0.778	0.769	-	0.158 –0.163	0.239 –0.244	0.239 –0.244	0.408 –0.414
**Dpw**	0.174	0.186	−0.114	0.108	0.065	0.161	-	0.564 –0.569	0.563 –0.569	0.049 –0.053
**Dpw'**	0.346	0.346	−0.152	0.151	0.076	0.242	0.566	-	1–1	0.099 –0.112
**Rao's D**	0.346	0.346	−0.152	0.151	0.076	0.242	0.566	1	-	0.098–0.112
**Rao's H**	0.283	0.11	−0.364	0.344	0.47	0.411	0.051	0.11	0.11	-

The lower triangle cell values are Pearson's *r* values being calculated from the Phylomatic phylogeny. Values in the upper triangle are 95% confidence intervals of *r* values calculated from the 100 randomly resolved phylogenies.

**Table 4:** Results of a principal components analysis of the eight phylogenetic beta diversity metrics used in this study.

	PC1	PC2	PC3
**D_pw_**	−0.041	−0.004	0.225
**D_pw_'**	−0.106	0.047	0.624
**D_nn_**	−0.415	−0.078	−0.294
**D_nn_'**	−0.55	−0.143	0.215
**PhyloSor**	−0.487	−0.285	−0.125
**UniFrac**	−0.369	−0.235	−0.092
**Rao's D**	−0.106	0.047	0.624
**Rao's H**	−0.358	0.912	−0.118
**Proportion of Variance Explained**	0.789	0.095	0.078
**Cumulative Variance Explained**	0.789	0.884	0.962

The loadings of the first three principal component (PC) axes are provided. The proportion of the variance explained by each axis and the cumulative variance explained are also provided.

## Discussion

The present study examined whether the species and phylogenetic beta diversity of tropical tree communities increased more with spatial or environmental distance. Specifically, I calculated the species and phylogenetic beta diversity of 96 tree inventory plots in India and asked whether this beta diversity was best predicted by the geographic distance, altitudinal difference or annual precipitation difference between the plots. The results of the Mantel tests show that species and phylogenetic beta diversity was most strongly correlated with differences in the annual precipitation between plots (Table 2). The geographic distance between plots and the difference in altitude between plots were weakly or not correlated with species and phylogenetic dissimilarities. This suggests that the abiotic environment, rather than space *per se*, plays a larger role in structuring the tree communities analyzed in this study. Given that the majority of the metrics employed are designed to detect ‘basal’ turnover of communities the results suggest that turnover along the precipitation gradient is in the form of turning over of clades and not species within clades. This suggests that species preferences with respect to precipitation are likely very dissimilar between distantly related species causing a pattern of phylogenetic turnover between communities along precipitation gradients. Though more detailed analyses into the entire distributions of the species studied, their functioning and null modeling analyses would be needed to substantiate this inference.

Additionally, for some phylogenetic metrics the correlation between the phylogenetic beta diversity and annual precipitation differences were stronger than those found using metrics of species beta diversity. This suggests that perhaps the phylogenetic metrics are detecting community structuring that could not be detected using a traditional species-centric approach. A traditional species-centric approach does take into account deeper phylogenetic relationships. It therefore cannot differentiate between the relatively little phylogenetic beta diversity presented by the spatial turnover of con-geners versus the relatively large phylogenetic beta diversity presented by the spatial turnover of species from different families for example. Therefore those cases where the phylogenetic beta diversity was more strongly related with the precipitation gradient than the species beta diversity suggests that phylogenetic measures of beta diversity have the ability to provide further information into the factors structuring communities.

The degree to which phylogenetic beta diversity metrics will help researchers understand the factors underlying community structure depends largely on how ecological strategies or traits evolve. In particular, phylogenetic investigations of communities have traditionally utilized the assumption that phylogenetic relatedness can be utilized as a proxy for ecological or trait similarity. If this assumption is supported then patterns of phylogenetic beta diversity should mirror the actual pattern of ecological or trait dissimilarity between communities. The validity of the assumption that there is phylogenetic signal in trait data is routinely questioned with researchers who suggest that it should be directly quantified rather than assumed in order to make robust inferences [3], [17], [18]. If statistical tests show significant phylogenetic signal in trait data then, it may be reasonable to assume that the phylogenetic patterns of beta diversity mirror the patterns of trait beta diversity.

In the present study, I evolved functional trait datasets with varying degrees of phylogenetic signal on the phylogenetic tree. These data were used to determine whether strong phylogenetic signal in trait data allowed for a mirroring of phylogenetic and functional beta diversities. When using the *PhyloSor*, *UniFrac* and nearest neighbor metrics I found that indeed the correlation between phylogenetic and functional dissimilarities was highest when there was more phylogenetic signal in trait data (Figure 2). In particular, when trait datasets had a *K* value greater than two the phylogenetic beta diversity was strongly correlated with the functional beta diversity suggesting that the phylogenetic measure was a solid proxy. When *K* values were less than one, predicting the functional beta diversity from the phylogenetic beta diversity was intractable. Thus when phylogenetic signal in trait data was high, these four metrics may generally be used to infer the functional beta diversity of communities.

In contrast to the above results, the degree in phylogenetic signal in trait data played a lesser role in whether the pairwise metrics and the Rao metrics of phylogenetic beta diversity mirrored the patterns of functional beta diversity. In other words, even when phylogenetic signal in trait data was high, these metrics often failed to serve as strong predictors of the functional beta diversity of communities. The failure of these metrics to recover the functional beta diversity is likely due to one to many factors, but here I will suggest just one. The trait and phylogenetic distance matrices are necessarily generated using different techniques. One could generate the trait distance matrix using a clustering method or Euclidean distance, but neither would be utilized when inferring phylogenetic trees. The difference in these methodologies alone may unlink the relationship between phylogenetic signal in raw data and phylogenetic and functional diversity relationships when using metrics that are ‘basal’ or pairwise. The influence is likely to be much reduced when only examining nearest phylogenetic or functional neighbors where the degree of incongruence in the distance matrices is much reduced.

Thus caution should be taken when attempting to infer the functional beta diversity of communities using pairwise metrics even when phylogenetic structure in trait data is detected. It is also worth noting that the present study utilized only one imaginary trait. Investigations that use multiple traits that have contrasting patterns of beta diversity may make inferences from phylogenetic beta diversity alone difficult. Such scenarios have been shown in measures of phylogenetic and trait diversity within individual communities [25].

The final goal of this study was to determine the statistical independence of the phylogenetic beta diversity metrics. As researchers have become more interested in quantifying the phylogenetic beta diversity of communities, the number of metrics has started to grow. During this growth phase it is likely that many similar metrics will be proposed. The present study has found that some metrics are identical or nearly identical suggesting that reporting just one of those metrics is sufficient. For example, the *PhyloSor* and *UniFrac*, metrics are nearly identical despite the latter being in the literature three years prior to the publication of the former [12], [15]. This should be obvious from an examination of the equations for each metric where both effectively incorporate all branch lengths connecting the species in two communities making them two sides of the same coin. Thus utilization of the original metric, *UniFrac*, should be sufficient. The nearest neighbor metrics fall into the same class of metrics as *UniFrac* and *PhyloSor* largely because they ignore the basal part of the phylogeny and only looks at nearest neighbor distances. The *UniFrac* and *PhyloSor* metrics utilize the basal parts of the phylogeny, but the majority of the variability in those metrics is to do with terminal relationships because in most cases the local communities sample most major basal lineages of the phylogeny. In other words the *UniFrac* and *PhyloSor* metrics generally saturate the basal parts of the phylogeny such that only the terminal turnover can vary and therefore drives the variability in these metrics. Furthermore metrics like *D_pw_*' and *Rao*'*s D* are also expected to be equivalent based solely upon how they are calculated. Both utilize a pairwise phylogenetic distance weighted by abundance. This is therefore another clear example of either authors not being aware of contemporary metrics or not comparing their ‘new’ metric to known existing metrics first either via simulation or by comparing their equations. As the number of metrics continues to grow, further studies will be needed to show which new metrics actually provide novel information and strengthen the statistical toolkit of the phylogenetic community ecologist.

The present study has shown that the phylogenetic beta diversity in a series of 96 India tree inventory plots is best predicted by a precipitation gradient rather than space. Thus the structure and turnover of communities in this system is phylogenetically non-random. Future tests are needed to determine what are the mechanisms underlying this non-random pattern. I have also shown that several phylogenetic metrics can correctly infer the functional beta diversity of communities when phylogenetic signal in trait data is high, while pairwise metrics often fail to do so. Lastly, I have shown that several metrics of phylogenetic beta diversity are largely redundant suggesting that only a few are needed to represent the breadth of the phylogenetic patterns in the system.

## Methods

The community composition data used in this study come from a network of 96 forest plots in the Western Ghats of India [26]. The plots are 1 ha in area and include all individual trees and lianas ≥10 cm in diameter 1.3 m above the ground. A total of 61,965 individuals and 446 species are contained in the database. The plots span two degrees of latitude (13.2°–15.2°), 1000 m in altitude (55 m–1060 m) and 7500 mm in annual rainfall (776 mm–8340 mm) making them ideally suited for analyses of community beta diversity.

A phylogenetic tree was generated to represent the 445 species in the forest plot dataset. This was accomplished using the informatics tool Phylomatic [27]. Branch lengths were assigned to the phylogeny using the informatics tool ‘bladj’ using the software Phylocom (www.phylodiversity.net/phylocom/). The bladj algorithm placed estimated node ages from Wikstrom et al. [28] onto the phylogenetic tree. Ages for nodes in the phylogeny without dates were then estimated by equally distributed ages between two nodes with ages. It should be noted that these age estimates are quite crude, but they provide a substantial improvement over setting all branch lengths to one (i.e. taxonomic beta diversity).

The phylogenetic tree generated for this study had a large number of terminal nodes left unresolved. The influence of resolution is an important issue in community phylogenetics with some studies seeking to directly estimate the bias this lack of resolution introduces [29], [30] and with others seeking to simply sequence all taxa in large diverse communities to generate resolved molecular phylogenies [29], [31]. In this study sequencing all taxa was not an option, so an alternative approach was utilized to estimate bias due to polytomies. The phylogenetic tree produced by Phylomatic was randomly resolved using Mesquite 100 times. The metric comparisons and the beta diversity correlations with space and the environment were all performed again with these 100 trees to provide an estimate of the range of possible values.

The present study used the eight metrics of phylogenetic dissimilarity described in the introduction. Additional metrics will undoubtedly be published in the near future, but at present this list composes the majority, if not the entirety, of the metrics available. I also calculated a Jaccard Distance and Bray-Curtis Distance between all forest plots using the R package ‘vegan’. The species and phylogenetic beta diversity values calculated were compared to the geographic, altitudinal and annual precipitation differences between the 96 forest plots using Mantel tests. Linear regression and principal components analyses were used to examine the statistical relationships between the dissimilarity metrics used in this study. Prior to performing the principal components analysis all outputs were normalized and transformed to dissimilarities, instead of similarities, to allow for comparison.

In order to quantify the degree to which phylogenetic signal in trait evolution influences the ability of different metrics of phylogenetic beta diversity to correctly infer the functional beta diversity I generated trait datasets with differing levels of phylogenetic signal. Specifically, using the R package ‘ape’, traits were evolved onto the phylogenetic tree using an Ornstein-Uhlenbeck model of trait evolution and differing levels of selective constraint. The selective constraint (i.e. alpha) values ranged from 0.2 to 0.99 and the optimal value (i.e. theta) was set to zero. A default value of sigma (0.1), the standard deviation of the random component for each branch, was used in all simulations. An alternative method for generating the trait datasets that does not require altering the selective constraint parameter would be simply to alter the values of sigma. This range of values was used in order to generate simulated trait datasets with a broad range of phylogenetic signal. Simulated datasets that maximized the range of *K* values possible were selected in order to explore a broader parameter space. The choice to use of varying levels of selective constraint in an Ornstein-Uhlenbeck model was made to generate variable trait datasets and was not meant to reflect whether or not this model is a better or worse estimate of how traits truly evolve in real systems. The phylogenetic signal in these trait datasets was measured using the *K* statistic of Blomberg et al. [32]. Values of *K* exceeding one generally indicate high phylogenetic signal in the trait data. Values of *K* below one indicate less phylogenetic signal in the trait data. The 60 trait datasets generated had *K* values ranging from ∼0.20 to 8.00.

The trait datasets were used to construct trait dendrograms using hierarchical clustering. These dendrograms representing the trait similarity of the tree species as is often done in studies that measure functional alpha diversity [33] and they provide the benefit of having data structures similar to phylogenetic trees making them easily analyzed by the same suite of beta diversity metrics. The functional beta diversity was calculated between each of the 96 forest plots using each of the beta diversity metrics. This was repeated using each of the 60 trait datasets. The functional beta diversity was then compared to the observed phylogenetic beta diversity using a linear regression. The R^2^ of that regression was then plotted against the *K* value of the trait dataset to determine how differing levels of phylogenetic signal in the trait data influenced the ability of the phylogenetic metrics to reflect the functional dissimilarity of communities.
